# Optical Detection of Vapor Mixtures Using Structurally Colored Butterfly and Moth Wings

**DOI:** 10.3390/s19143058

**Published:** 2019-07-11

**Authors:** Gábor Piszter, Krisztián Kertész, Zsolt Bálint, László Péter Biró

**Affiliations:** 1 Institute of Technical Physics and Materials Science, Centre for Energy Research, P.O. Box 49, H-1525 Budapest, Hungary; 2 Hungarian Natural History Museum, 13 Baross St., H-1088 Budapest, Hungary

**Keywords:** butterfly wing, moth wing, photonic crystal, vapor sensing, vapor mixture, principal component analysis, chemical selectivity, optical readout

## Abstract

Photonic nanoarchitectures in the wing scales of butterflies and moths are capable of fast and chemically selective vapor sensing due to changing color when volatile vapors are introduced to the surrounding atmosphere. This process is based on the capillary condensation of the vapors, which results in the conformal change of the chitin-air nanoarchitectures and leads to a vapor-specific optical response. Here, we investigated the optical responses of the wing scales of several butterfly and moth species when mixtures of different volatile vapors were applied to the surrounding atmosphere. We found that the optical responses for the different vapor mixtures fell between the optical responses of the two pure solvents in all the investigated specimens. The detailed evaluation, using principal component analysis, showed that the butterfly-wing-based sensor material is capable of differentiating between vapor mixtures as the structural color response was found to be characteristic for each of them.

## 1. Introduction

The efficient detection of volatile organic compounds (VOCs) in current applications is highly needed as monitoring air quality in the living environment is becoming increasingly important [1,2,3,4,5]. For the characterization of VOCs in the ambient atmosphere, inexpensive sensors, which combine chemically selective detection with high sensitivity, are required. The multivariate sensors, based on photonic crystals, are excellent candidates for this task as their optical readout is relatively easy and their response time is fast, while they can operate in ambient air without high operating temperature or vacuum conditions [6,7]. Even though human-made photonic crystals have been available for more than 30 years [8], the low-cost and large-scale production of these intricate structures has not yet been implemented. The photonic nanoarchitectures provided by nature [9,10] are cheap and ready-made nanostructures produced in macroscopic quantities and can instantly be used in various applications [1]. These structures can be found in plants [11,12,13], insects [14,15], aquatic animals [16,17], and birds [18,19,20,21]. The photonic nanoarchitectures in the wing scales of butterflies [22,23], insect cuticles [24], and the barbules of bird feathers [25] were found to exhibit a measurable optical response when the vapor composition of the ambient atmosphere changed. Therefore, they can be used as sensor materials for vapor detection with an optical readout. This color change was shown to be fully reversible [22] because the open structure of these porous photonic nanoarchitectures allows fast exchange with the ambient vapors [26]. The vapor-induced color changes were also found to be substance-selective in many types of photonic nanoarchitectures [27,28]. Based on these examples, artificial bio-inspired sensor materials were developed [6,7,29].

**Figure 1:**
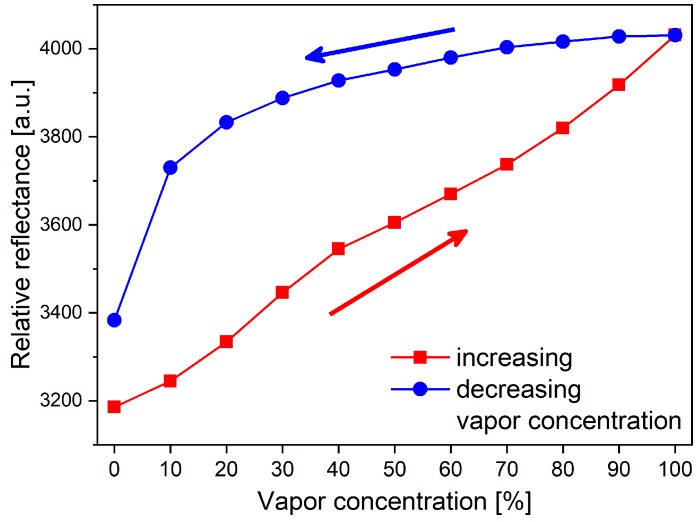
Peak intensity of the relative reflectance spectrum of a *Polyommatus icarus* specimen during increasing/decreasing ethanol vapor concentration. The graph showing hysteresis suggests that capillary condensation of the vapors is the governing process during the color change of the butterfly wings.

In our previous research, we investigated the vapor-sensing properties of nine closely related polyommatine blue butterfly species in which the males have structural colors on their dorsal wing surfaces [30]. These structural colors are generated by quasi-ordered three-dimensional photonic nanoarchitectures that are composed of a chitin matrix with embedded air holes and can be found in the lumen of the wing scales [31]. During vapor exposition, the optical responses of these structures are based on the capillary condensation of the vapors into the nanoarchitectures, which was confirmed by the measured hysteresis during increasing/decreasing vapor concentrations (Figure 1) [28,32]. Due to the condensed liquid layer, swelling of the chitin nanoarchitecture occurs [27], which enables both the higher-than-expected optical response [22] and the material-specific behavior during the vapor exposition [28]. These processes result in a relatively high sensitivity and provide chemically selective vapor sensing [28]. The vapor-sensing properties of the wing scales can also be modified [28,33]. Using atomic layer deposition on the wing scales, the optical response was significantly reduced, showing that vapor-induced color change originates from the combination of the refractive index change caused by the condensed vapors and the swelling of the chitin matrix [28]. Using liquid pretreatment, the sensitivity of the butterfly-wing-based vapor sensor was more than doubled and the selectivity in the low vapor concentration regime was significantly enhanced [33]. These experiments and other experiments which were reported in the literature, were conducted on whole butterfly wings using a bench-top optical setup. However, vapor sensing can be performed not only on whole wings, but also on single separated scales [34]. The optical responses of single scales were also shown to be vapor-specific using a microscope-based setup, and the sensitivity of the single scales could be enhanced by stacking several scales onto each other [34].

The sensors based on biological photonic nanoarchitectures can be considered multivariate sensors, which means that they demonstrate a high response dimensionality in the detection of condensable vapors [1,6,7]. This is reflected in the chemically selective behavior, suggesting that they could be used to efficiently analyze vapor mixtures of different VOCs. In this study, we investigated for the first time how the structural color changes in a wide range of photonic nanoarchitectures occurring in butterfly and moth wings when mixtures of different vapors were applied in the surrounding atmosphere, and how the optical response developed during this vapor-mixture exposure. To show the efficiency of the discrimination of the butterfly wings, we first used water and acetic acid vapor mixtures in different concentrations and dilutions, and by using principal component analysis (PCA) on the measured data, we showed that the increasing acetic acid concentration shifts the trajectories in the principal component (PC) scores plot but preserves the initial orientation and monotonic behavior of the trajectories, which demonstrates that it is possible to discriminate between the acetic acid solutions with 5% concentration differences in all dilutions. To test the chemical selectivity, different vapor mixtures were also prepared from individual gas bubblers by mixing the vapors using digital mass flow controllers. These volatile mixtures were tested on several butterfly species with structural coloration and we found that the relative reflectance spectra (structural color change) of the mixtures always fell between the optical responses of the two pure solvents in all the investigated specimens. In the case of *Hypochrysops polycletus*, detailed analysis was conducted on three different ethanol vapor mixtures using PCA and we found that each mixture produced differently directed trajectories in the PC scores plot, which showed that the butterfly wing-based sensor material is capable of distinguishing between different vapor mixtures, as the structural color response was found to be characteristic in all three cases.

## 2. Materials and Methods

**Figure 2:**
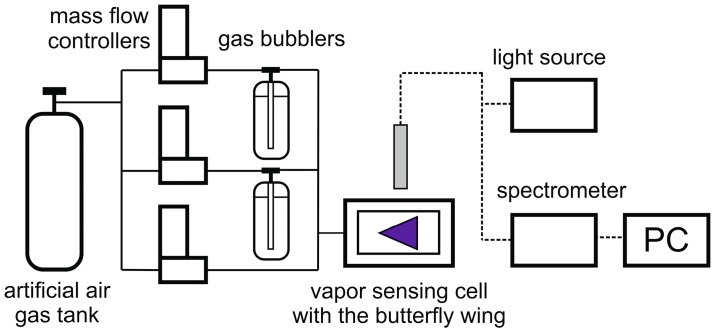
Schematic drawing of the measurement setup used in the vapor sensing experiment.

The investigated species were not subjected to any restriction. The samples used in this study belonged to the curated collection of the Hungarian Natural History Museum. The males of *Heliophorus yunnani* (Lycaenidae: Heliophorini) [35], *Morpho aega* (Nymphalidae: Morphini) [23,36], *Polyommatus icarus* (Lycaendiae: Polyommatini) [30,37], *Chrysiridia rhipheus* (Uraniindae) [23,38], and *Hypochrysops polycletus* (Lycaenidae: Luciini) [39] species all possess photonic nanoarchitectures in their dorsal wing scales which generate intense structural colors. These open nanostructures allow for the fast exchange of test substances during vapor exposure, which results in high vapor-induced optical responses of the wings. The separated wings were inserted into an air-proof aluminum cell with a gas inlet and outlet (Figure 2). During the vapor sensing experiment, a constant gas flow of 1000 mL/min was used [28]. The saturated vapors for the mixtures were obtained from two gas bubblers and the vapor concentration was set by computer-controlled digital mass flow controllers (Aalborg DFC, Aalborg Instruments & Controls Inc., Orangeburg, NY, USA), which mixed the saturated vapors and the synthetic air to the desired ratio. At every vapor concentration step, a 20 s vapor flow was followed by a 60 s purge of synthetic air to recover the initial reflectance of the wings before the introduction of the next mixture. The optical responses of the wings were measured through the quartz window of the vapor sensing cell, which provided transmittance in the whole ultraviolet-visible (UV-VIS) wavelength range. The samples were illuminated by an Avantes DH-S-BAL (Avantes BV, Apeldoorn, Netherlands) deuterium-halogen light source under normal incidence, while the reflected light was collected under ~45°, where the highest intensity of the wing reflectance was detected. The reflectances of the wings were measured with an Avantes HS 1024×22TEC spectrophotometer (Avantes BV, Apeldoorn, Netherlands). The optical response of the wings during vapor exposure can be characterized by the color change of the photonic nanoarchitecture. To express this color change signal, the relative reflectance spectra (∆***R***) were introduced [22], where the initial spectrum of the wings in artificial air (***R_0_***) was used as a reference during the measurements: ∆***R*** = (***R***/***R*_0_**) × 100%.

All the substances (acetic acid, acetone, ethanol, and 2-propanol) used in the vapor sensing measurements are reagent grade and were obtained from VWR International Ltd. (Radnor, PA, USA).

The data processing of the measured reflectance spectra and the principal component analysis were conducted using OriginLab OriginPro 2018 software (OriginLab Co., Northampton, MA, USA).

## 3. Results

**Figure 3:**
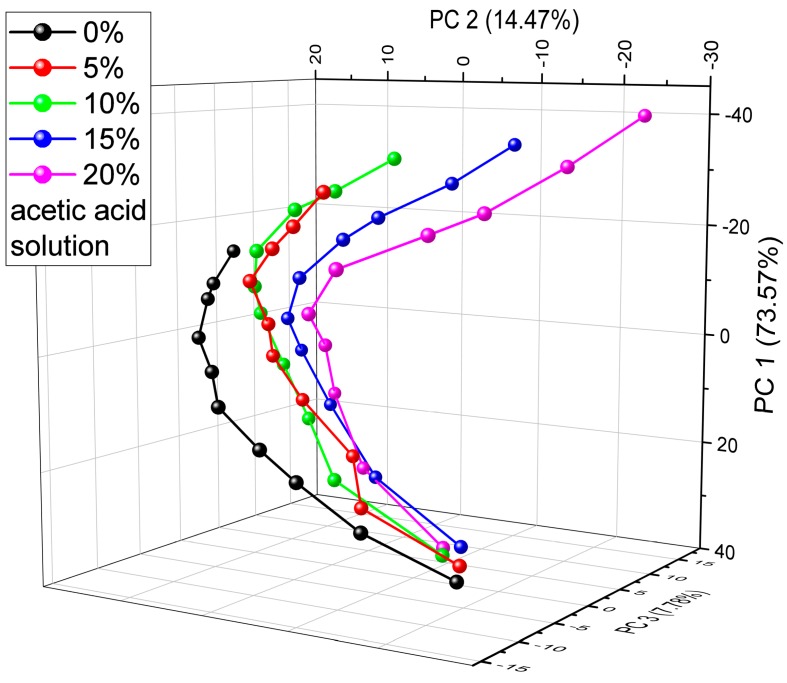
Principal component analysis (PCA) scores plot of the first three principal components (PCs) of the vapor sensing dataset measure on the dorsal wing surface of a male *P. icarus* specimen. Distilled water (0%) and four acetic acid solutions were applied (5–20%) in the gas bubblers to prepare the vapors in the desired concentrations. The vapor concentration increases from the bottom of the graph to the top from artificial air to saturated vapor mixtures.

The optical response of the wings of a male *P. icarus* specimen was investigated when the vapor mixtures of two different materials (water, acetic acid) were introduced into the sensing cell. We investigated how the structural color changed during the exposure of mixtures of different concentrations and how it is related to the previous results for the pristine vapors. In our previous research, we found that in the PCA results of the vapor sensing measurement with seven test substances, the trajectories of water and the 10% acetic acid solution were close to each other, showing their similar optical effects when they were applied [28]. Here, we prepared four different acetic acid and water solutions, where the acetic acid content ranged from 5% to 20%, and applied them as test substances in the vapor sensing experiment. The optical responses were measured for every solution at 10 concentration values from 0% (pure artificial air) to 100% (saturated vapor of the solution). The reflectance spectra measured during the vapor exposure were analyzed using PCA. The PC scores plot of pure water (0%) and the four solutions (5–20%) in Figure 3 shows that the vapor-specific trajectories shifted with increasing acetic acid concentration, but they preserved the characteristic shape throughout the whole concentration range. The vapor concentration along the trajectories increases from the bottom to the top of the plot. The cumulative variance of the PCs was 96%; PC 1 = 73.57%, PC 2 = 14.47%, PC 3 = 7.78%.

**Figure 4:**
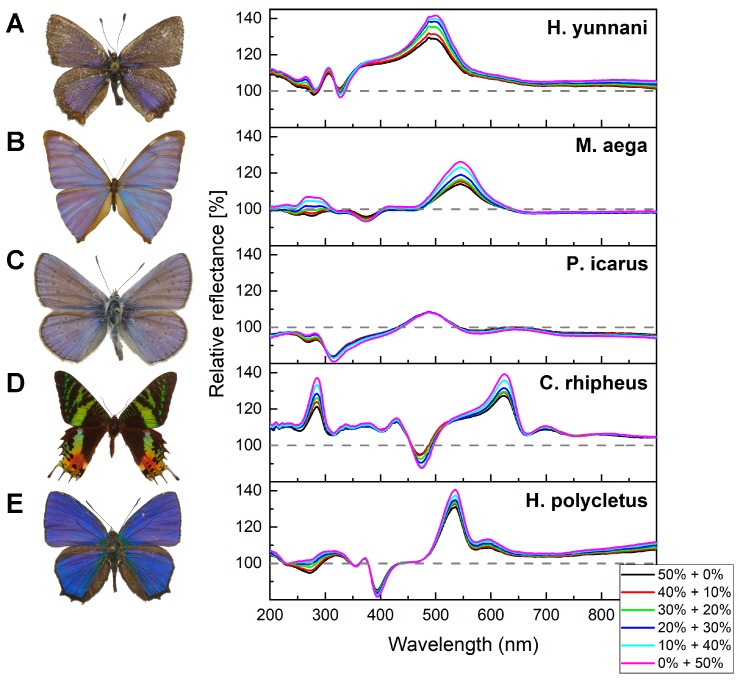
Optical responses of the wings of five species (photographs are not to scale) were investigated when mixtures of acetone and ethanol vapors were applied. (**A**) *H. yunnani*, (**B**) *M. aega*, (**C**) *P. icarus*, the green parts of (**D**) *C. rhipheus*, and (**E**) *H. polycletus* all showed similar color change behavior: the optical responses of the mixtures fell between the color change signals of the pure solvents. The broken lines show the initial relative reflectance spectra used as a reference (100%).

Due to the highly different volatilities of the investigated substances, the preparation of the vapor mixtures from the liquid solutions was not possible in all cases. Therefore, for all the used mixtures, we applied two separate gas bubblers with pure substances and the so-produced saturated vapors were mixed together in the desired ratio with digital mass flow controllers (Figure 2). A third channel was used, providing pure artificial air to dilute the vapor mixtures to the desired extent. A disadvantage of this method is that we could not explore the whole concentration range of the vapor mixtures, as, at small flow rates, the gas bubblers did not work properly. Therefore, only higher vapor concentrations were applied, where the ratio of the artificial air was less than 50% in the mixture. Nevertheless, it was possible to investigate the optical responses of different wings when the concentration of the components was changed in the mixtures. Figure 4 displays the optical responses of five species when mixtures of acetone and ethanol vapors were applied with 50% artificial air dilution.

**Figure 5:**
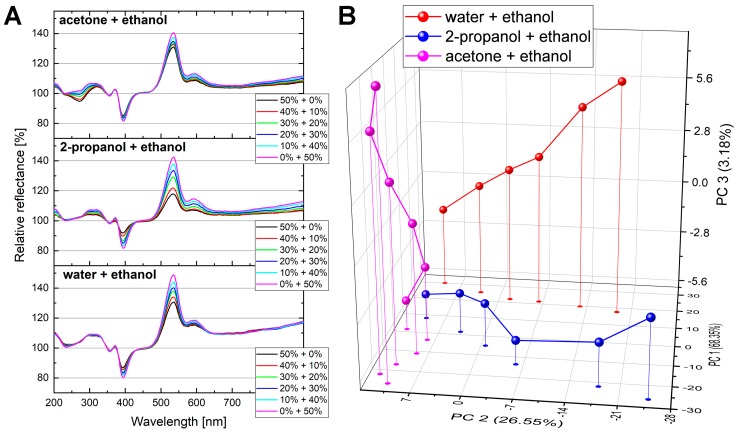
Different vapor mixtures generated different optical responses of the wing of a *H. polycletus* male specimen. Acetone + ethanol, 2-propanol + ethanol, and water + ethanol mixtures were applied. The concentration of the first saturated vapor component was set from 0% to 50%, while the second component was set adversely (50% to 0%). The whole mixture was also diluted with artificial air in a 1:1 ratio. (**A**) The optical responses (relative reflectances) of the three mixtures are characteristically different from each other. (**B**) The measured relative reflectances were analyzed using PCA. The PC scores plot shows the characteristic trajectories of the three mixtures. The three points close to each other on the left side are the points of the pure ethanol vapor from which the trajectories spread apart.

To investigate the chemically selective behavior of the butterfly-wing-based sensor in detail, a *H. polycletus* male specimen was used in the vapor sensing experiment and three different vapor mixtures were applied. The measured optical responses, i.e., the reflectance change spectra of the wing in the case of acetone + ethanol, 2-propanol + ethanol, and water + ethanol vapor mixtures were recorded, and the results are depicted in Figure 5A. The concentration of the first saturated vapor component was set from 0% to 50%, whereas the second component was set adversely (50% to 0%). The mixture was diluted with artificial air in a 1:1 ratio. All vapor mixtures showed similar behavior, as described earlier, as the optical responses of the mixtures fell between the pure solvents. To analyze the vapor-specific behavior, the measured data were evaluated with PCA. Figure 5B provides the PC scores plot, which contains the vapor sensing trajectories of the three different vapor mixtures. The cumulative variance of the PCs was 98%; PC 1 = 68.35%, PC 2 = 26.55%, PC 3 = 3.18%. The three data points on the left that are close to each other, from which the trajectories originate, are the points of the pure ethanol vapor spectrum. Away from this, the curves spread apart, as the compositions of the mixtures shift to their other components (water, 2-propanol, and acetone).

**Figure 6:**
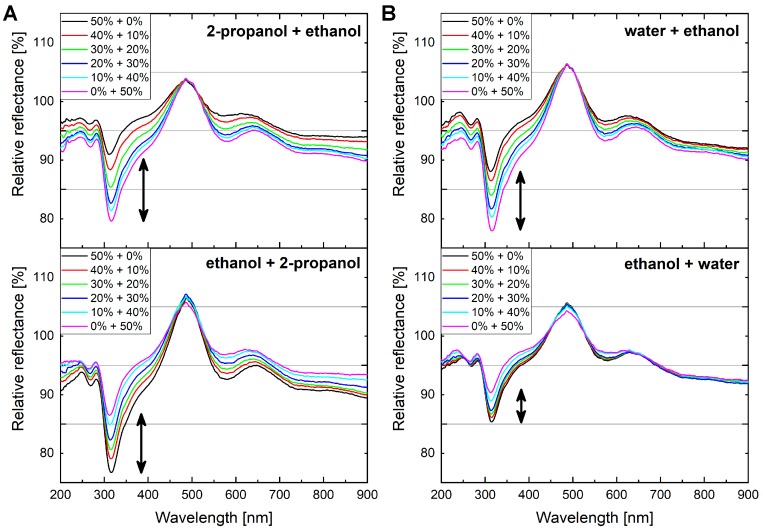
Optical responses of the wings of a *P. icarus* male specimen when 2-propanol + ethanol and water + ethanol vapor mixtures were applied twice, but with the second in reverse order. (**A**) 2-propanol + ethanol vapor mixtures were applied in reverse order without any issue. (**B**) Water + ethanol vapor mixtures show a significant memory effect compared to the reverse order measurement.

The effect of vapor mixtures applied in reverse order was also investigated. We used a *P. icarus* male specimen as the sensor material and applied two different vapor mixtures: 2-propanol + ethanol and water + ethanol. In this case, we used the same protocol that was introduced in the last section: a 1:1 ratio of the vapor mixture and artificial air, and the concentration of the vapor mixture components set between 0% and 50%. After the first measurement, a five-minute-long purging with artificial air was applied and a second measurement was recorded where the vapors were applied in reverse order. Figure 6 depicts the results of the two measurements. When the two volatile components, 2-propanol and ethanol, were applied (Figure 6A), the butterfly wing showed highly similar optical responses, despite using the reverse order. However, when ethanol and water vapors were mixed together (Figure 6B), high asymmetry was observed between the two optical responses conducted in reverse order, showing the memory effect of the sensor for water vapors.

## 4. Discussion

The ambient atmosphere in our living environment can be considered a mixture of gases and vapors. To monitor air quality, i.e., the composition of such a complex system, multivariate sensors are required to efficiently detect the gas or vapor contents of this elaborate mixture. Sensors based on structurally colored wings were shown to be an effective tool for the detection of condensable vapors [6,22,23], but the investigation of vapor mixtures in ambient air is still in its infancy [40]. Here, the optical responses of several butterfly and moth species to vapor mixtures were investigated. The blue wing scales of *P. icarus* male specimens contain quasi-ordered photonic nanoarchitectures that can effectively detect vapors of different concentrations through capillary condensation and swelling of the chitin nanoarchitecture [28]. As the different vapors caused substance-specific optical responses [28], we expected unique and specific color changes in the case of vapor mixtures. Figure 3 depicts the PC scores plot of the different acetic acid solutions applied as test substances. In our previous research, the trajectories of water and 10% acetic acid solution were close to each other and had a similar shape [28]. Here, we found that the trajectories of the different acetic acid solutions appeared in a consecutive order from pure water to a 20% concentration solution and retained their initial shape, showing that an increasing acetic acid content shifted the optical response of the butterfly wing scale and that this shift was proportional to its concentration in the solution. Due to the concentration-dependent shift, all the measured optical responses were specific to a certain mixture and to a specific vapor concentration: the increase in the acetic acid concentration in the 0%–10%–15%–20% series shifted the end point of the trajectory in a roughly linear dependence and caused the monotonic extension of the overall length of the trajectory in the figure. The 20% acetic acid concentration approximatively doubled the length of the trajectory. Chitin exhibits substance-specific swelling [41]; therefore, this regular modification of the shape of the trajectories is attributed to the enhanced swelling induced by the increasing amount of acetic acid in the vapor mixture. In the case of the 5% and 10% solutions, the trajectories overlap slightly, which may be due to the low acetic acid content of the former mixture.

The vapors prepared from pre-mixed solutions provided promising results, but, in most cases when the components’ volatilities are significantly different, a separate preparation of the vapors is needed. To achieve this, two independent gas bubblers with the required substances were used, and the saturated vapors were mixed together in the desired ratio using mass flow controllers. This mixture was also diluted with artificial air to control the concentration of the vapor mixture. With this setup, we investigated the optical responses of five structurally colored Lepidoptera species. Figure 4 depicts the characteristic responses for acetone + ethanol mixtures. The changes in the structural colors caused by the different acetone + ethanol vapor mixtures always fell between the optical responses generated by the two pure substances in a consecutive order, i.e., the phenomenon that was demonstrated in detail with acetic acid + water solutions occurred in all investigated species and also with other substances. The optical response from a certain vapor mixture consists of the linear combination of the optical responses caused by the vapor components separately. As long as characteristic differences exist between the two components’ optical responses (e.g., wavelength shifts in the relative reflectance spectra), the optical response of the vapor mixture will also be substance-specific. This suggests that the photonic nanoarchitectures occurring in the wing scales are capable of chemically selective sensing of pure vapors as well as vapor mixtures. The different sensitivities of the different photonic nanoarchitectures were also observed. The relative reflectances of *M. aega*, *C. rhipheus*, and *H. polycletus* specimens during the vapor exposition showed a high degree of separation in the high ethanol concentration range (20% acetone + 30% ethanol, 10% acetone + 40% ethanol, and 0% acetone + 50% ethanol), whereas in the case of *H. yunnani*, we observed equidistant increases throughout the entire concentration range. In this measurement, the wing of *P. icarus* only showed moderate sensitivity and a significant color change was only observed in the near-UV wavelength range.

To demonstrate the chemically selective sensing of the vapor mixtures, a butterfly species with an intense structural color and a photonic nanoarchitecture exhibiting a long-range order (photonic crystal-type structure) was selected. The wings of a *H. polycletus* male specimen were tested with three different vapor mixtures that all contained ethanol as one component. The mixtures of saturated vapors were diluted with artificial air to avoid condensation of the vapors into the sensing cell. In Figure 5A, the characteristic optical responses caused by the different vapor mixtures show substance-specific behavior. The signal of pure ethanol vapor (magenta curves in Figure 5A) has a similar shape in every case, representing the reproducibility of the measurement; the other components’ signals were significantly different from each other, representing the vapor sensor’s chemically selective behavior. The PC scores plot of the same measurement (Figure 5B) shows that the optical response evolved as the vapor mixtures’ composition shifted from pure ethanol to the other substances. The vapor sensing trajectories originated from a common point (pure ethanol) and these curves spread apart with decreasing ethanol content, showing that the optical response changed as the other components were added. Each of the tested substances that were added to ethanol generated trajectories parallel to one of the planes of the three-dimensional (3D) representation in Figure 5, and each added substance defined a different plane in this 3D representation. These results demonstrate that a butterfly wing-based sensor material can discriminate between different vapor mixtures, as the optical response of these biological photonic nanoarchitectures is substance-specific.

Finally, in the case of aqueous solutions, a so-called memory effect may occur. In Figure 6, the results of two independent measurements are depicted, where the order of the components was reversed in the second case. When two volatile compounds were applied, no differences were observed, but when water was used instead of the first component, a slight alteration in the measured optical response occurred. This is not an irreversible color change of the wing, but longer purging times with artificial air are required when water vapors in higher concentrations are used. This is attributed to the less volatile nature of water compared to other substances, whereas its interaction with the chitin photonic nanoarchitecture is relatively strong, which also results in high optical responses.

## 5. Conclusions

Multivariate sensors demonstrate a high-response dimensionality in the detection of condensable vapors, which is reflected in their chemically selective behavior. Biological photonic nanoarchitectures, occurring in the wing scales of butterflies and moths, were found to be efficient multivariate sensors, which was manifested in their substance-specific optical responses [1,6,7,22,23,28]. In this study, the vapor sensing capabilities of five butterfly and moth species were investigated when mixtures of VOCs were applied in the surrounding atmosphere. It was found that optical responses of the mixtures are the linear combination of the optical responses of the pure solvents, showing that the biological photonic nanoarchitecture-based sensor materials can differentiate between vapor mixtures, as the optical response was found to be characteristic for each of them. Further PCA evaluation confirmed the chemically selective behavior, as the trajectories in the PC scores plot were found to be differently directed when the second components were added to ethanol vapor. When mixtures based on water vapor were applied, significant memory effect occurred, which can be attributed to the less volatile nature of water compared to the VOCs. It is important to investigate how water vapor affects the optical response signal because this is the highest concentration vapor in ambient air. In our future experiments, we will focus on simulating real-life conditions and investigate how water-based vapor mixtures affect the substance-selective vapor sensing of structurally colored wings. Furthermore, the dynamic features of the optical response during the exposition of vapor mixtures can be analyzed using machine learning algorithms. This new approach would provide additional information on the interaction between the nanoarchitectures and the tested substances, which could allow us to discriminate between vapor mixtures of three or more components.
